# Early outcomes of colon laparoscopic resection in the elderly patients compared with the younger

**DOI:** 10.1186/1471-2482-12-S1-S8

**Published:** 2012-11-15

**Authors:** Vincenzo Bottino, Maria Grazia Esposito, Arianna Mottola, Giampaolo Marte, Vittorio Di Maio, Valerio Sciascia, Marco Nunziante, Giovanni Fregola, Salvatore Cuzzovaglia, Francesco Galante, Federica Andreoli, Alfredo Breglia, Maria Elena Giuliano, Domenico Papaleo, Paola Della Rocca, Pietro Maida

**Affiliations:** 1 Evangelic Hospital Villa Betania, Via Argine 604, Naples, Italy

## Abstract

**Background:**

The aim of this study was to define any benefits in terms of early outcome for laparoscopic colectomy in patients over 75 years old (OP) compared with the outcomes of a younger populations (YP).

**Methods:**

Forty elderly patients undergoing laparoscopic colectomy for colorectal cancer between 2007-2011 were studied, the patients are divided for gender, age, year of surgery, site of cancer, and comorbidity on admission and compared with 40 younger patients.

**Results and discussion:**

Mean (standard deviation) age was 81.3 in OP and 68.3 YP Conversion rate was the same between the two groups. There was no difference in operative mean time . The overall mortality rate was 0% percent. The surgical morbidity rate was the same but there was an increased in cardiologic e bronchopneumonia complications in older population. Patients treated with laparoscopic approach had a faster recovery of bowel function and a significant reduction of the mean length of hospital stay not age related. Laparoscopy allowed a better preservation of postoperative independence status.

**Conclusions:**

Laparoscopic colectomy for cancer in elderly patients is safe and beneficial including preservation of postoperative independence and a reduction of length of hospital stay.

## Background

Life expectancy is increasing along with the number of elderly patients with surgically correctable diseases. Recent reports suggest that age *per se* in the absence of significant disease should not be considered a prognostic factor in gastrointestinal surgery [1]. Associated comorbid conditions, in particular, pulmonary and cardiovascular, are mainly responsible for the higher morbidity and mortality rate reported in the very elderly patient [2]. A substantial amount of evidence indicating that conventional surgery in elderly patients with colorectal cancer is well tolerated and results in similar survival curves compared with younger counterparts seem to support the aforementioned findings [2-5]. The appropriate approach to surgical intervention in these patients is a major issue. Minimally invasive surgery has been proven to reduce hospitalization, postoperative pain, and cardiopulmonary stress, to better preserve immune and metabolic responses, and to allow a more rapid return to their routine activities [6-8]. These findings suggest that the laparoscopic approach should appear to be the ideal surgical choice for elderly patients.

The aim of the present study was to define any benefits in terms of early outcome in patients 75 years old or more who underwent laparoscopic colectomy (LPS) for cancer compared with the a younger population.

## Methods

A prospective registry of all patients undergoing a colon or rectal procedure has been maintained in our department since 2007. Demographics, biochemistry values, nutritional status, operative variables, comorbidity factors on admission, postoperative outcome, and histopathology of more than 300 patients are currently recorded. Comorbidity factors on hospital admission were assessed according to the American Society of Anesthesiologists (ASA) score [9]. Nutritional status was defined according to body mass index (BMI) and weight loss greater than 10 percent (in the six months before hospital admission). This study included 40 patients 75 years old or more who were candidates for elective laparoscopic colon or rectal resection from January 2007 to December 2011. Exclusion criteria were emergency surgery, presence of a fixed palpable mass, and cancer infiltrating adjacent organs. Forty eligible subjects underwent laparoscopic colorectal resection after they signed an informed consent form. Each laparoscopy patient was matched with a younger control patient who had undergone laparoscopic surgery identified from the institution’s database from January 2007. The same exclusion criteria adopted for elderly cases were used in the selection of controls. Controls were selected to match for site of primary disease, year of surgery, gender and when was possible, nutritional status.

In all patients bowel preparation was performed the day before the operation by intestinal washout with an iso-osmotic solution (3 litres). For antibiotic prophylaxis all patients received a dose of Unasyn (3 g intravenously) during the induction of anaesthesia. A second dose of the same antibiotic was administered if surgery lasted more than four hours. Deep vein thrombosis prophylaxis was performed with low-molecular-weight heparin (3000 IU/day) in all patients. Postoperative infusion of fluids and electrolytes was given to all patients according to clinical requirements. Postoperative oral feeding did not differ between older and younger patients. Clear liquid was started between postoperative days 4 and 5, as tolerated by the patient. The laparoscopic procedure was performed according to our technique and is always the same. Conversion to open surgery was defined as an abdominal incision longer than 10 cm or an abdominal incision different from that planned at the start of the operation.

The following details of the surgical procedure were recorded for all patients: duration of operation, operative blood loss, and amount of homologous blood transfused. Tumour classification was by TNM stage. The number of lymph nodes collected in the specimen was also recorded for all patients.

Postoperative bowel function was evaluated with respect to the first flatus and bowel movement (measured in days). In all patients progressive recovery of oral feeding began after first flatus. Microbiologic analysis and positive culture showed all infectious complications. Clinically evident anastomotic leak was also recorded. Registration of complications and the need for a new hospitalization were recorded for the first 30 postoperative days. Moreover, at the one-year follow-up, patients were monitored for complications and hospital readmissions by office visit or phone interview.

## Results and discussion

The demographics and clinical characteristics of the patients enrolled in the study. There were 40 patients in the LPS group and 40 patients in the open group. Mean (SD) age was 81.3 (2.3) years in the OP group and 68.0 (3.1) years in the YP group. The two groups were well matched for demographics and nutritional variables. Among patients with cancer of the rectum, the mean (SD) distance of the tumour from the anal verge was 7 (3.1) cm in the OP group and 6 (3.4) cm in the YP group (Table 1).

**Table 1:** Demographics and clinical characteristics of the two groups

Variable	old (n = 40 patients)	young (n = 40 patients)
Age (yr)	81.3 (2.3)	68 (3.1)

Gender M/F	20/22	20/22

ASA score	3,1 ± 0.1	2.1 ± 0.6

BMI	25.01 (2.8)	24.11 (2.9)

Hemoglobin (g/liter)	11.7 (2.0)	12.0 (1.6)

Weight loss (>10%)	10	7

Cancer stage (TNM)		

I	8	8

II	8	10

III	20	19

IV	4	3

Type of operation		

Right hemicolectomy	12	12

Left hemicolectomy	5	4

Sigmoid resection	13	14

Rectal resection	10	10

Data are number of patients or mean.

No difference was found with respect to TNM cancer stage and type of operation performed. The mean number of lymph nodes intraoperatively collected was 18.2 (8.8) in the OP group and 18.7 (7.8) in the YP group (*P* = 0.74).

In 1 patient (2.5%) in the OP group and 1 patient in the YP, conversion to open surgery was necessary in the first case for adhesion in the second for narrow pelvis. There was no conversion for laparoscopic complications.

Operative variables are listed in Table 2 The mean operative time was nearly the same. Mean operative blood loss was the same. (Table 2)

**Table 2:** Intraoperative variables in the two groups

Variable	OP (n = 40 patients)	YP (n = 40 patients)
Operative time	220.27 (58.2)	215.2 (48.6)

Operative blood loss	135 (115)	135 (115)

No. of transfused patients (%)	5/40 (12,5%)	2/40 (5%)

LPS = laparoscopic.

^a^Only in transfused patients.

The overall mortality rate was 0%. The reoperation rate was 5% (2/40 patients) in the OP group and 2.5% (1/40 patients) in the YP group. There was nearly no difference with respect to the type of postoperative complications in the two groups except for a greater incidence of pulmonary and cardiac complications in OP group.

Patients in both groups have experienced an earlier mean canalization, a faster recovery of bowel function 4.8 (2.1) days and mean length of hospital stay 9.8 (5.3) days compared with the outcomes of conventional open surgery.

The mean time of follow-up was 24.7 (median, 22; range, 12–55) months. Analysis of complications that occurred later than 30 days after surgery was censored at one year after operation. At the time of complications analysis, there were 35 patients alive in the OP group and 39 in the YP group. Complications occurred in three patients OP (two intestinal obstruction, one incisional hernia on previous trocar site) and in two patients YP (one intestinal obstruction, one incisional hernia on previous trocar site. Hospital readmission was necessary for two OP patients (intestinal obstruction) and for one patients in the YP group (intestinal obstruction). (Table 3).

**Table 3:** Number of patients with postoperative complications in the two groups

Variable (%)	OP (n = 40 patients)	YP (n = 40 patients)
Overall	12 (30.%)	10 (25%)

Infectious ^a^	8(20.%)	5 (12.5%)

Noninfectious ^a^	3(7,5.%)	4(10%)

Anastomotic leak ^a^	1 (2.5%)	1(2,5%)

LPS = laparoscopic.

^a^Numbers of single type of complication do not add up to the number of overall complications within the two groups because of the possible occurrence of more than one type of complication in some patients.

Studies reporting the early outcome after laparoscopic colorectal resection in elderly patients have been published in the literature [11-16,26,27], but some studies were lacking proper controls and only a few papers considered cancer patients only [12,15,16,27]. Moreover, in our study patients were matched for the site of primary disease and the operations performed were homogeneous in the two groups, avoiding the bias of unbalanced operations.

Our findings support the hypothesis that laparoscopic surgery in the elderly is safe and stress the fact that age *per se* in the absence of significant disease should not be considered a prognostic factor in gastrointestinal surgery. In our study there was a low conversion rate and no conversion was a result of intraoperative complications. The low conversion rate and the absence of intraoperative complications caused by the minimally invasive technique reported here may reflect adequate training of the surgical team and a strict selection policy, which mandates the exclusion of patients with locally advanced disease.

In the OP group the overall morbidity rate was 30%, which is comparable with other studies of laparoscopic colectomy in elderly patients and is consistent with the results of studies in general population. In particular, we found a different incidence ( more in the OP) of both cardiac and pulmonary complications but if we compare OP with old people treated with traditional approach (open surgery) there is no difference. These findings are consistent with the pooled rate reported by Abraham *et al*. in a systematic review of randomized trials comparing the short-term outcome after laparoscopic resection with open resection for colorectal cancer. These findings are noteworthy and suggest that the laparoscopic technique could be safely used in elderly patients who seem to tolerate well the hemodynamic and ventilatory changes observed in laparoscopic surgery, the longer operation time, and the frequent steep head-down tilt (Trendelenburg position) which are usually required during a laparoscopic operation. All the aforementioned variables have been previously reported to influence intraoperative and postoperative morbidity rate in high risk patients [19][20].

In this study the overall morbidity rate was not statistically different in the two groups.

The analysis of operative variables confirmed that the laparoscopic operation in OP was no longer and there is no difference in blood loss compared with the same operation in younger people

There was no difference in hospital stay, it was 9.8 days. As reported by others for elderly patients [14,22,23,25] length of hospital stay for laparoscopy patients was the same compared with the Younger patients, Similar findings were reported by Senagore and colleagues who found no difference between patients 70 years old or older who underwent laparoscopic colectomy compared with patients younger than 60 years [23,26,27]. The shorter length of hospital stay observed in the LPS group could be ascribed to the earlier recovery of bowel function and to the better recovery to full independence. Other factors that could influence the duration of hospital stay are less postoperative pain and analgesic consumption, a lower postoperative complication rate, and an earlier recovery of full ambulation activity [21,23]. Using a multimodal rehabilitation protocol, Badram and colleagues reported a median postoperative stay of 2.5 days for patients with a median age of 81 years who had undergone laparoscopic colonic resection. However, they reported a high readmission and reoperation rate, which could affect the independence rate in these critically ill patients [21,22].

The significantly lower need for post hospital nursing observed in the LPS group deserves major consideration, in particular for high-risk patients, such as octogenarians, because of quality of life and financial implications.

## Conclusions

Laparoscopic colorectal surgery in people older than 75 years is safe, offers the same short-term benefits that younger individuals have, a shorter length of hospital stay, an earlier recovery of bowel function, and a higher rate of postoperative independence at discharge.

## Abbreviations

LPS: laparoscopic; ASA: American Society of Anesthesiologists; BMI: body mass index; TNM: Tumor Node Metastasis staging; OP: older population; YP: youger population; LPS: Laparoscopic.

## Competing interests

The Authors declare that they have no competing interests.

## Authors' contributions

VB, VDM, PM: conception and design,interpretation of data, given final approval of the version to be published; MGE, AM, GM, VS, MN, GV, SC, FG, FA, AB, MEG, DP, DRP: acquisition of data, drafting the manuscript, given final approval of the version to be published.
